# Stanford type A aortic dissection after urgent prosthetic valve replacement: case reports

**DOI:** 10.1186/1749-8090-9-9

**Published:** 2014-01-07

**Authors:** Yoshihiro Suematsu, Sei Morizumi, Kenichi Okamura, Mitsuhiro Kawata

**Affiliations:** 1 Department of Cardiovascular Surgery, Tsukuba Memorial Hospital, Tsukuba, Japan

**Keywords:** Aortic valve replacement, Aortic dissection, Aortic dilatation, Urgent surgery

## Abstract

Occurrence of acute aortic dissection after aortic valve replacement is rare, however, it is associated with high mortality and morbidity rates. We report two Asian cases in which acute aortic dissection occurred after urgent aortic valve replacement for infective endocarditis. Successful graft replacement was carried out with preservation of the prosthetic valves in both cases. Our experience with these cases suggests that, even in urgent or emergent situations, surgical intervention for associated aortic dilatation should be considered when aortic valve replacement is performed.

## Background

Although occurrence of acute aortic dissection after aortic valve replacement (AVR) is rare, however, it is associates with high mortality and morbidity rates [1,2]. Herein, we present two cases in which acute aortic dissection occurred after aortic valve replacement for infective endocarditis, in which successful grafting was carried out with preservation of the prosthetic valves.

### Case 1

**Figure 1:**
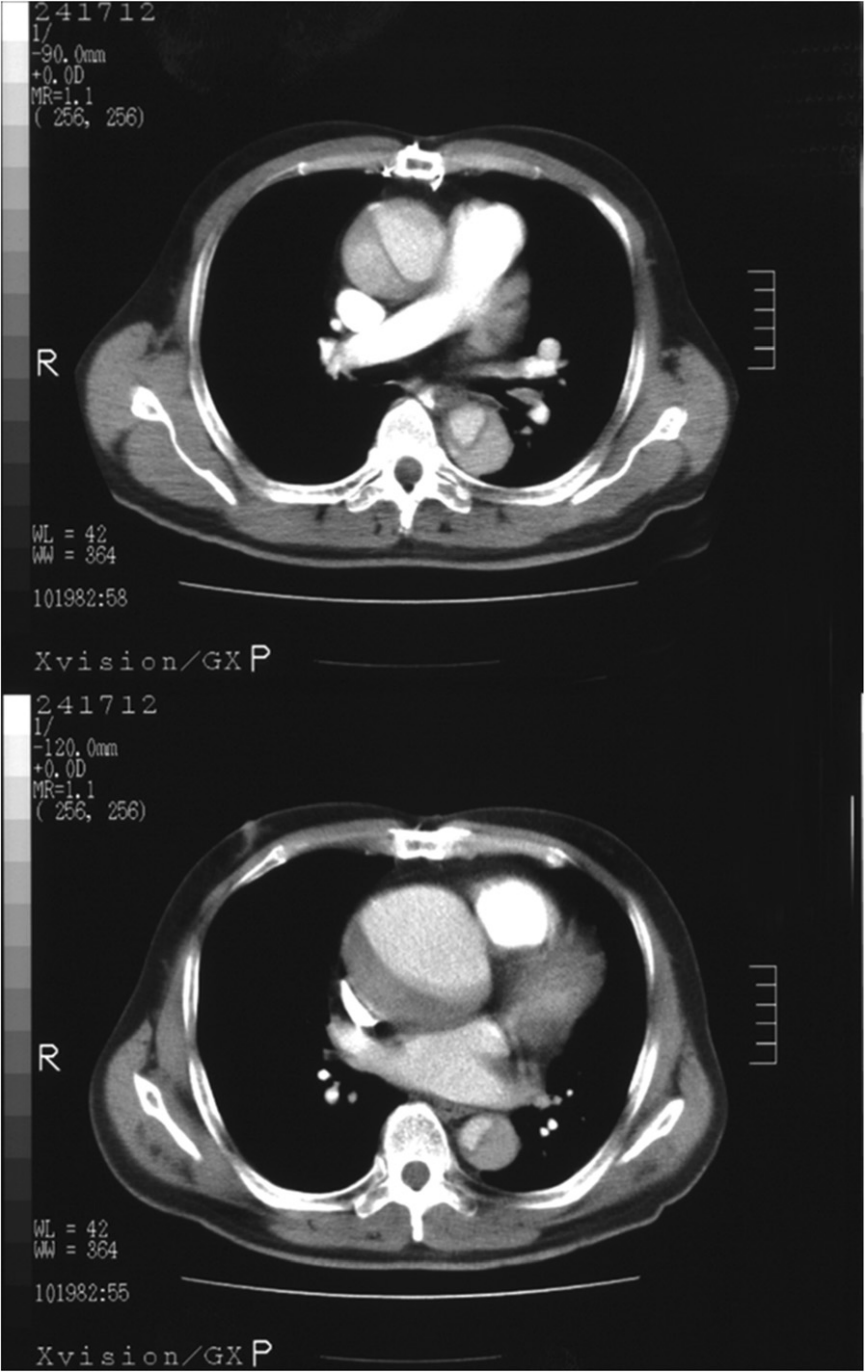
Computed tomography angiography (CTA) showing dissection of the ascending aorta.

The patient was a 57-year-old Asian male who underwent treatment for dental caries in March 2004. Two months later, he developed high fever and visited a nearby hospital. He had only a history of hypertension and no contributory family history, including of Marfan syndrome. Echocardiography revealed moderate aortic and mitral valve insufficiency with obvious vegetations. The ascending aorta was enlarged in width to 44 mm. Blood culture failed to isolate any bacteria. Roth’s spots were detected in the eyes, and Osler’s nodes in the fingers of the right hand. Under the clinical diagnosis of infective endocarditis, the patient was initiated on intravenous antibiotic therapy with Penicillin G and gentamicin. However, he failed to show satisfactory response to the medical therapy, the symptoms of heart failure deteriorated, and he developed several distal embolic episodes. Urgent double valve replacement (Aortic valve: ATS #25 mm, Mitral valve: ATS #31 mm) was performed. The native aortic valve was tricuspid. The ascending aorta was left untouched at this time-point. Examination of the operative samples from the mitral and aortic valves revealed bacterium and eumycetes. Therefore, administration of Fosfluconazole, meropenem, and vancomysin was started. The postoperative course was uneventful and the infection was well-controlled. The patient was discharged and followed up at the outpatient clinic thereafter. Four years later, in January, 2008, the patient presented with chest pain of sudden onset, and computed tomography angiography (CTA) showed dissection of the ascending aorta (Stanford A) (Figure 1). A re-do sternotomy was undertaken immediately, and cardiopulmonary bypass was established with arterial perfusion from the femoral artery and bicaval drainage. The entry was located in the ascending aorta, away from the previous cannulation and aortotomy sites. The mechanical valve at the aortic position was preserved. Aortic root and ascending aortic replacement with retrograde cerebral perfusion in deep hypothermia (18°C) and reconstruction of the right and left coronary arteries was performed. Histopathological examination did not show any evidence of inflammation caused by bacteria, but only atherosclerotic changes of the vessel wall without medial necrosis. At present, 5 years later, the patient is doing well.

### Case 2

**Figure 2:**
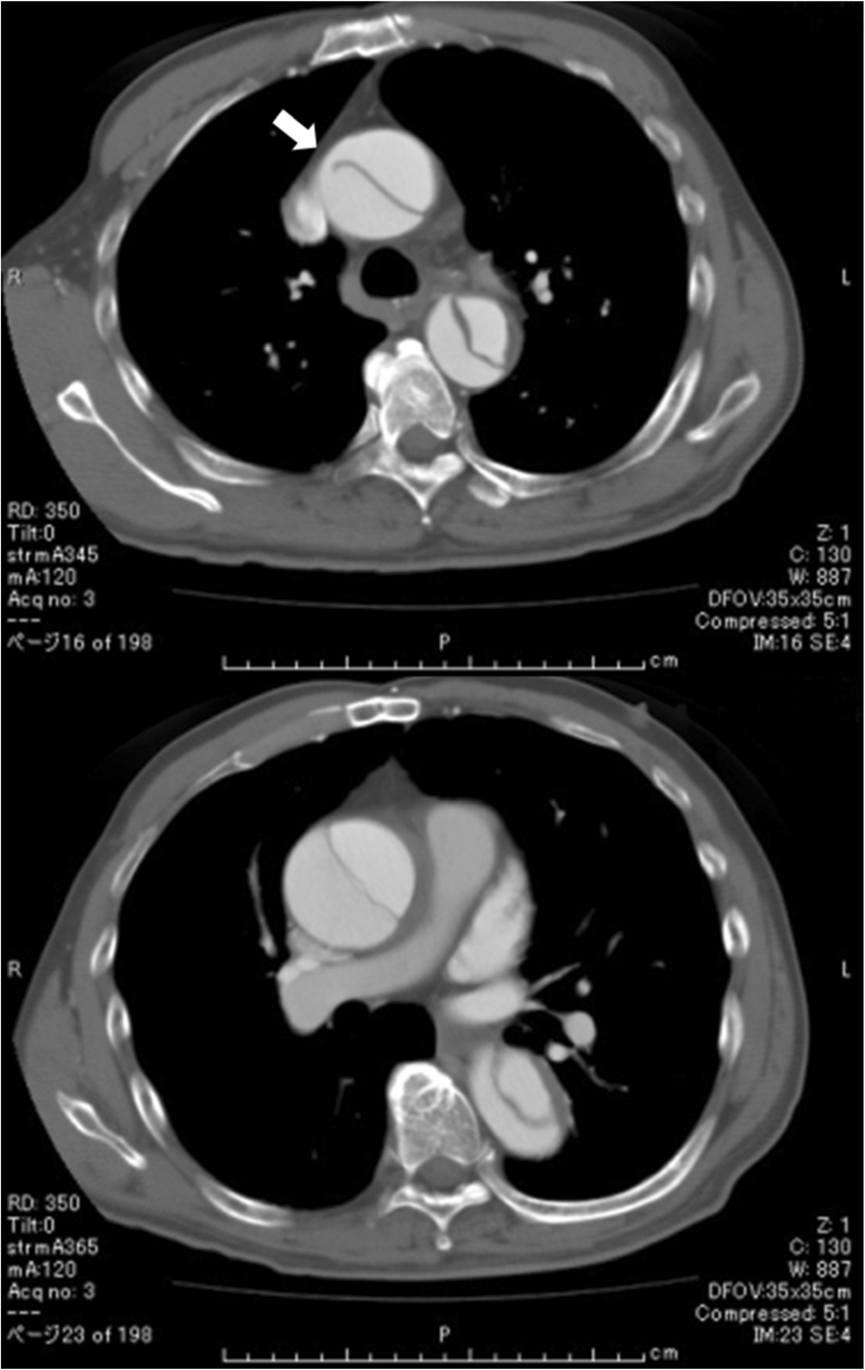
CTA showing intimal tear (arrow) and dissection of the ascending aorta.

**Figure 3:**
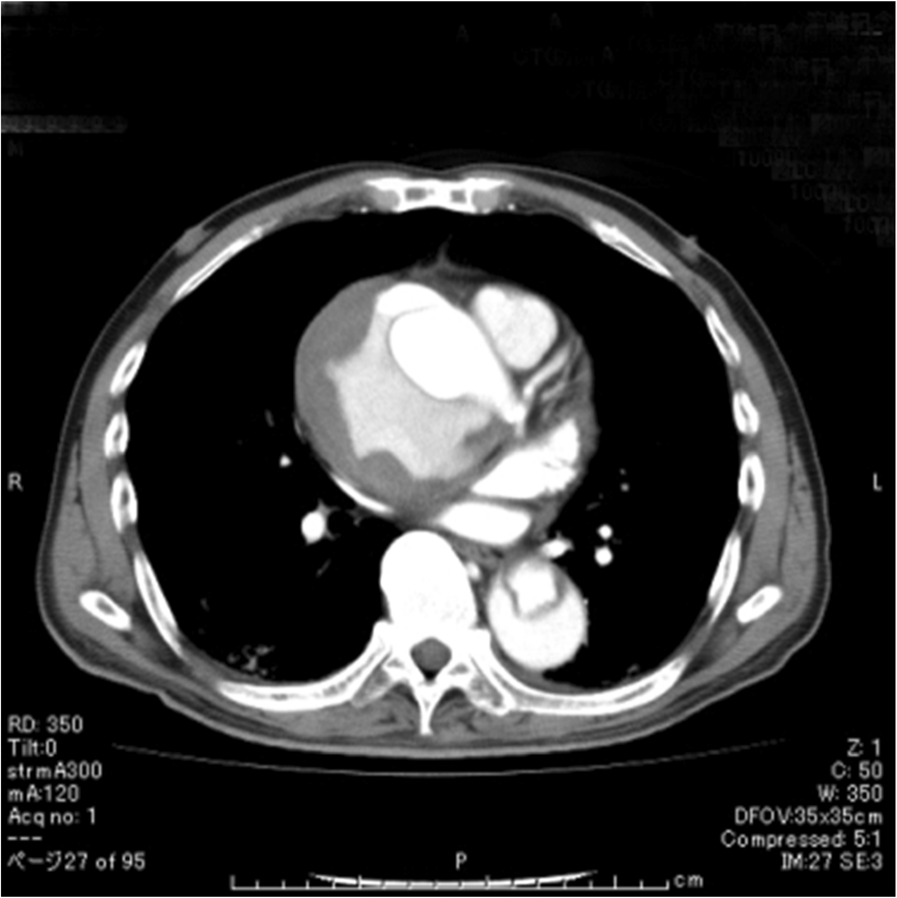
CTA showing enlargement of the ascending aortic width to 80-mm.

The second patient was a 62-year-old Asian male, who complained of fever and dyspnea on exertion in January 2000. He had no significant past medical history or any contributory family history. He went to a nearby hospital and echocardiography revealed moderate aortic valve insufficiency with vegetations on the valve and dilatation of the ascending aorta to 46 mm. The patient was initiated on antibiotic therapy and during the course of the hospitalization for the therapy. However, because of repeated embolic episodes, urgent aortic valve replacement (Carpentier-Edwards tissue valve: #25 mm) was performed. The native aortic valve was tricuspid. Nine years later, in March 2009, the patient presented with severe right leg pain, and was admitted to the hospital. The right femoral arterial pulse was not palpable on arrival. A CTA showed dissection of the ascending aorta continuing to the iliac bifurcation and obstruction of the right common iliac artery (Stanford A) (Figure 2). Initially, we performed surgical fenestration and cross-over femoral bypass grafting with an 8-mm PTFE graft for the right leg ischemia, since the patient and his family refused consent for open heart surgery. Fortunately, there was no pericardial effusion and no evidence of aortic bioprosthetic valve insufficiency. The postoperative course was uneventful. However, one month later, a repeat CTA showed gradual enlargement of the ascending aortic width to 80-mm (Figure 3). Therefore, we persuaded the patient and his family to provide consent for surgery on the ascending aortic aneurysm. Cardiopulmonary bypass was established with arterial perfusion from the femoral bypass graft and bicaval drainage after re-sternotomy. The entry was located in the ascending aorta, away from the previous cannulation and aortotomy sites. The false lumen extended to the right coronary ostium and just above the annulus. A re-do aortic valve replacement was not considered necessary as there was no prosthetic valve deterioration. Ascending aortic replacement with retrograde cerebral perfusion in deep hypothermia (18°C) and right coronary artery reconstruction were performed. Similar to the findings in case 1, histopathological examination did not show any evidence of inflammation caused by bacteria, but only atherosclerotic changes of the vessel wall without medial necrosis. The immediate postoperative course was uneventful and the patient was discharged three weeks after the second surgery. At present, 4 years since the surgery, the patient continues to do well.

## Case presentation

Acute aortic dissection after aortic valve replacement is rare, estimated to occur at a rate of 0.53-2.3%. The reported average interval from initial surgery to aortic dissection ranges from several days to over 10 years [1,2]. The presumed cause of this condition is manipulation of the aorta during the initial surgery [3]. Previous aortotomy or manipulations such as aortic perfusion, cardioplegia of the root, cross-clamping, etc. can cause intimal tears. On the other hand, while Fukuda et al. [4] reported that the common factor in all of his 6 reported patients was cystic medial necrosis of the aorta. However, histopathological examination of the excised aorta in our patients did not reveal any evidence of cystic medial necrosis.

Some of the possible causes of aortic dilatation prior to the AVR are infected aortic aneurysm or infective aortitis. Various microorganisms have been reported to be associated with this condition, most commonly staphylococci, enterococci, streptococci, and salmonella species [5]. In our cases, the relationship between the preoperative aortic dilatation and infection remains unclear, however, histopathological examination did not show any evidence of inflammation caused by bacteria.

In a patient with marked enlargement of the aorta (>50 mm) at the time of the initial AVR, AVR plus ascending aortic replacement would be the gold standard. However, there is no consensus on whether a mildly dilated aorta (40-50V mm) should be surgically treated. Tsutsumi et al. [6] reported that aortic regurgitation combined with systemic hypertension, male sex, and a thinned or fragile aorta with mild dilatation (>45 mm) at initial AVR may be risk factors for late aortic complications. Albat et al. [7] reported that, in their series of 752 AVR cases, aortic dissection occurred in 0.53%, with the ascending aortic width being >55 mm at the time of the initial operation in 29% of cases. Based on their experience, they are performing prosthetic graft replacement for enlargement of the ascending aortic width to >55 mm, and systemic reinforcement with a Dacron mesh for enlargement of the ascending aortic width to 45-50 mm, concomitantly with the AVR. Both procedures have been shown to have long-term effectiveness. The results also appear reasonable from the point of view of consistent with LaPlace’s law. According to LaPlace’s law, the risk of further dilatation of the ascending aorta, aortic rupture, and aortic dissection definitely increases with increasing diameter of the ascending aorta. Even in the absence of structural damage of the connective tissue, graft replacement or size reduction surgery may be a promising approach.

However, most surgeons hesitate to perform AVR plus ascending aortic replacement in elderly patients who have poor cardiac function, because prolonged extracorporeal circulation may lead to cerebral edema, respiratory dysfunction, acute renal failure, and myocardial damage, which can increase the morbidity and mortality.

Currently, based on these experiences, we perform aortic plication with reinforcement using Teflon felt for enlargement of the ascending aortic width to 40-50 mm, concomitantly with AVR, however, the issue of reinforcement or reduction ascending aorotoplication requires continued re-evaluation because there are some adverse reports such as “under-the-wrap” aortic wall atrophy [8].

## Conclusions

We report two cases in which acute aortic dissection occurred after aortic valve replacement for infective endocarditis. Successful graft replacement was carried out with preservation of the prosthetic valves in both cases. Our experience with these cases suggests that careful follow up is required for patients with a dilated ascending aorta, followed by valve surgery, and surgical intervention for the associated aortic dilatation should be considered, even in urgent or emergent situations, when aortic valve replacement is performed.

## Consent

“Written informed consent was obtained from the patients for publication of this case report and any accompanying images. A copy of the written consents is available for review by the Editor-in-Chief of this journal”.

## Abbreviations

AVR: Aortic valve replacement; CTA: Computed tomography angiography.

## Competing interests

The authors declare that they have no competing interests.

## Authors’ contributions

YS wrote the draft of the manuscript and obtained the written consent. SM, KO, and MK aided in literature search and gave final approval of the manuscript. All authors read and approved the final manuscript.
